# Some Nursing Developments

**Published:** 1916-02-12

**Authors:** 


### KING'S COLLEGE HOSPITAL.

### Some Nursing Developments.

The new hospital at Denmark Hill will afford accommodation for 600 patients, and is in some respects the most finely equipped institution for the sick in this country. One of its salient features from the nursing point of view is the full extent to which the requirements of the nursing staff have been met, both in the wards and in the nursing home. It represents an attempt to realise the soundest ideals of nursing administration and domestic science, to forestall the nurse's needs in respect to the patient's well-being, and to set the entire staff in the best possible relation to their work by removing every impediment to health and comfort. The result is an object-lesson which cannot possibly be grasped in its full significance without careful study. It may be said, indeed, that none but those actually engaged in the higher branches of nursing administration at the new King's are in a position fully to appreciate the extent to which the comprehensive ward scheme facilitates the work of the institution. To take in the purpose of every detail in the plan and act up to the standard thus visibly erected requires nursing ability and powers of organisation of an uncommon type. The main purport of the training-school is to develop this ability, so that the sisters brought into being under these admirable conditions may have learned the great secret of nursing, which is to bring the entire environment of the sick into conformity with the laws of hygiene as at present understood.

### The New Probationer.

There is no preliminary school. Suitable candidates, who must be between twenty-three and thirty years of age, serve a month on probation, and if then accepted sign an agreement for three years. For their first year's work they receive no pay, and they are liable to a forfeit of £5 if they leave before the conclusion of the term for which they have signed. During the first nine months of their training probationers go through a carefully thought out course of preliminary training. This includes classes in elementary anatomy and physiology, given by the assistant matron, and practical demonstrations in nursing, together with bandaging classes by one of the ward sisters. The course comprises such preparation as the preliminary school supplies in some hospitals before the probationer enters the wards, and is undertaken systematically with a view to making the probationer efficient as soon as possible in the discharge of elementary ward duties. It is, of course, of a far more thorough character than it is possible to give during a few weeks in a preliminary school, and it is believed that the progress of the probationer is facilitated by her admission from the first to participate in the duties for which she is preparing, without the break in continuity which is inevitable in the other plan. There is an excellent class and lecture room for the use of the nursing students, with many excellent models and charts. During this early period of their training the probationers are distinctly in the status of pupils, although their services are available under supervision in the wards. The examination which closes this period is decisive as regards the future of the probationer.

### Lectures and Classes.

Junior probationers attend classes given by the assistant matron on (1) sepsis, antisepsis, asepsis; (2) inflammation; (3) fractures; (4) burns; (5) germs; (6) wounds; (7) hæmorrhage; (8) instruments. The assistant matron also gives classes between November and June on anatomy, physiology, the nursing of certain diseases, bedsores, and other subjects. It may be mentioned that the assistant matron holds the position of nursing tutor in the training-school—that is to say, she controls the entire teaching course under the matron, supervises the note-taking and preparation for lectures, and prepares the students for their examinations.

Senior probationers attend lectures from the visiting staff of the hospital on medical and surgical nursing, on gynæcological nursing, on the nursing of diseases of the throat, the ear, and the eye, on the nursing of sick children, on the teeth, on hygiene, and on bacteriology. Every lecture in these interesting and varied courses is followed by a class held by the assistant matron. Written and *viva voce* examinations are held twice a year, and practical examinations once a year.

